# Clinical detection and monitoring of acute pulmonary embolism: proof of concept of a computer-based method

**DOI:** 10.1186/2110-5820-1-33

**Published:** 2011-08-11

**Authors:** James A Revie, David J Stevenson, J Geoffrey Chase, Christopher E Hann, Bernard C Lambermont, Alexandre Ghuysen, Philippe Kolh, Philippe Morimont, Geoffrey M Shaw, Thomas Desaive

**Affiliations:** 1 Department of Mechanical Engineering, Centre of Bioengineering, University of Canterbury, Christchurch, New Zealand; 2 Department of Electrical Engineering, University of Canterbury, Christchurch, New Zealand; 3 Cardiovascular Research Center, University of Liege, Belgium; 4 Department of Intensive Care, Christchurch Hospital, Christchurch, New Zealand

## Abstract

**Background:**

The diagnostic ability of computer-based methods for cardiovascular system (CVS) monitoring offers significant clinical potential. This research tests the clinical applicability of a newly improved computer-based method for the proof of concept case of tracking changes in important hemodynamic indices due to the influence acute pulmonary embolism (APE).

**Methods:**

Hemodynamic measurements from a porcine model of APE were used to validate the method. Of these measurements, only those that are clinically available or inferable were used in to identify pig-specific computer models of the CVS, including the aortic and pulmonary artery pressure, stroke volume, heart rate, global end diastolic volume, and mitral and tricuspid valve closure times. Changes in the computer-derived parameters were analyzed and compared with experimental metrics and clinical indices to assess the clinical applicability of the technique and its ability to track the disease state.

**Results:**

The subject-specific computer models accurately captured the increase in pulmonary resistance (*R_pul_*), the main cardiovascular consequence of APE, in all five pigs trials, which related well (R^2 ^= 0.81) with the experimentally derived pulmonary vascular resistance. An increase in right ventricular contractility was identified, as expected, consistent with known reflex responses to APE. Furthermore, the modeled right ventricular expansion index (the ratio of right to left ventricular end diastolic volumes) closely followed the trends seen in the measured data (R^2 ^= 0.92) used for validation, with sharp increases seen in the metric for the two pigs in a near-death state. These results show that the pig-specific models are capable of tracking disease-dependent changes in pulmonary resistance (afterload), right ventricular contractility (inotropy), and ventricular loading (preload) during induced APE. Continuous, accurate estimation of these fundamental metrics of cardiovascular status can help to assist clinicians with diagnosis, monitoring, and therapy-based decisions in an intensive care environment. Furthermore, because the method only uses measurements already available in the ICU, it can be implemented with no added risk to the patient and little extra cost.

**Conclusions:**

This computer-based monitoring method shows potential for real-time, continuous diagnosis and monitoring of acute CVS dysfunction in critically ill patients.

## Background

Traditional hemodynamic monitoring in critical care is constrained by the ease and frequency at which measurements can be made and taken. The use, number, and positioning of catheters is determined by the perceived risk-to-benefit ratio of inserting the device(s). Hence, catheters cannot always be placed in the most ideal position for monitoring a certain pathological state. Other factors, including expense and obstruction to clinical work flow, limit the regularity at which other measurements are taken, such as with the use of computer tomography and echocardiography. Due to these constraints, clinicians often do not receive a full picture of a patient's hemodynamic state, which can result in misdiagnosis and incorrect treatment, leading to inefficient use of hospital resources, increased length of stay, and death [1-5]. Integration of all available measurements in a mathematical framework of cardiovascular physiology could be employed to predict cardiac and circulatory status continuously in regions that cannot be directly measured. Such a method would provide a means to untangle the complex, often confusing interactions that occur between different measurements to better reveal a patient's underlying disease state, and therefore, maximize the useful information gained.

It has been proposed, and proven with glycemic control [6-8] and in other fields [9-12], that computer-based models can be used to assist medical staff with therapy-based decisions. For a model-based method to be clinically viable for hemodynamic monitoring in clinical practice it should:

• Accurately predict static markers of the cardiovascular health,

• Track pathologically important hemodynamic trends,

• Track the effectiveness of treatment,

• Be inexpensive and easy to implement,

• Provide continuous real-time feedback,

• Be an improvement on current hemodynamic monitoring methods.

**Figure 1:**
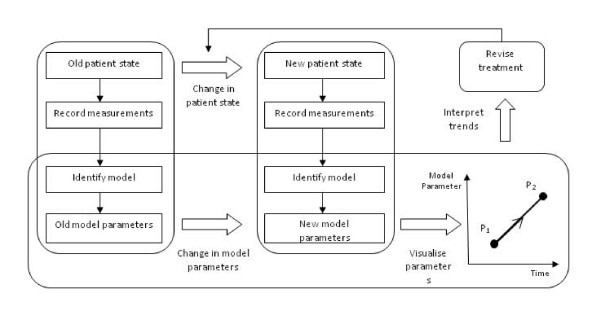
**Proposed usage of model-based monitoring techniques**.

If these points can be achieved, a better understanding of the patient's disease state can be obtained without added cost or invasive measurements, and the effectiveness of treatment can be monitored with greater accuracy, ultimately leading to improved outcome. A flow chart illustrating the proposed role of a model-based method in intensive care unit (ICU) is shown in Figure 1.

Previously, a mathematical model of the cardiovascular system (CVS) has been validated in different cardiac and circulatory states [13-16] and a method of personalizing the model from hemodynamic measurements had been created [17-20]. However, this method lacked clinical applicability for several reasons:

1) It required knowledge of the left and right ventricular volume waveforms

2) It was too slow to give real-time information

3) Either knowledge of the left and right ventricular pressure waveforms were required or population-based assumptions were needed to identify the valvular resistances

Hence, to truly individualize a computer model of the CVS to each patient, highly invasive or expensive measurements of the left and right ventricle's were required, limiting the clinical use of this method.

This paper presents initial results from a newly developed method [21,22], in which subject-specific modes of the cardiovascular system are identified from typically available ICU measurements. The object of the research was to test the accuracy and clinical applicability of this technology, with data from a previous study by Ghuysen et al. [23], before it is tested in human trials. This retrospective, proof of concept animal study uses measurements obtained from five porcine trials where acute pulmonary embolism (APE) was induced. The method has already been shown to predict static markers of CVS health, but its ability to monitor pathologically induced trends has not been assessed.

Hence, the goal of the paper was to assess the ability of the computer model to track clinically relevant hemodynamic changes resulting from APE. Changes in the model parameters are compared to clinically expected trends from the literature, experimentally derived metrics, and measured data, to test the ability of the model to track cardiovascular changes resulting from APE [23-25]. The overall goal was to demonstrate the clinical relevance and prove the concept (and potential) of model-based clinical monitoring of CVS status and acute dysfunction.

## Methods

A computer-based hemodynamic monitoring technique was retrospectively tested using measurements from a porcine model of APE. In this research, a mathematical model acts as framework to which metrics of cardiac and circulatory state can be personalized to describe the subject particular hemodynamic state. The CVS model is individualized to each pig using a model identification method, which matches the model to cardiovascular measurements form porcine trials. The pig-specific models are then compared to measurements not used to identify the models and experimentally derived indices to assess their accuracy and clinical potential.

### Cardiovascular system model

A lumped parameter model of CVS has been previously validated [13-16,19]. In this type of model the "lumped" parameters describe the macrodynamics of large sections of the CVS, instead of small localized effects. For example, the model parameter, systemic vascular resistance, represents the combined effects of the resistance of the all the vessels in the body, whereas localized differences in the resistance of small sections of the vasculature are not modeled. The lumped nature allows the model to be relatively simple, requiring only a small number of parameters, yet complex enough to describe all the important global hemodynamics of the CVS.

**Figure 2:**
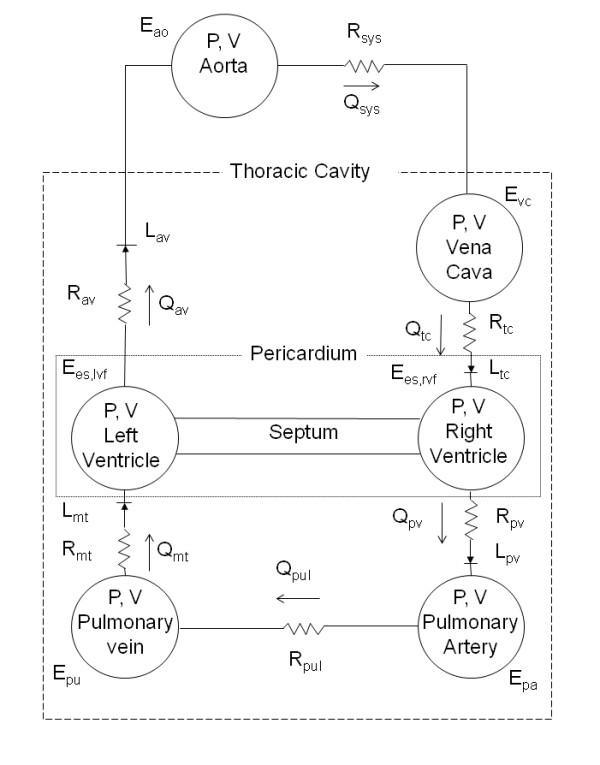
**Overview of the six-chamber cardiovascular model**.

The CVS model consists of six elastic pressure-volume chambers representing the left and right ventricles, aorta, vena cava, pulmonary artery, and pulmonary vein (Figure 2). Myocardium activation is modeled using time varying elastance in the left and right ventricle chambers, which act as the pumps for the model. In other words, time-varying elastance acts as a way of modeling the active increase in pressure in the ventricles resulting from cardiac contraction. The other chambers are passively elastic, and they react to changes in volume by proportionally changing pressure. The flow between chambers is controlled by a series of resistances. These resistances define what the pressure differences between adjacent chambers are for a given flow rate and physically describe how difficult it is to push blood through certain parts of the CVS. Flow-gated inertial heart valves close to avoid backwards flow and describe the inertial effects of the fast-flowing blood through the valves. Furthermore, the interdependence of the ventricles is modeled through septum and pericardium dynamics. The combination of these model features and parameters provide a means for accurately depicting ventricular, arterial, and venous blood pressures and volumes, and the blood flow rates through the heart valves, body, and lungs. A full list of the model equations, parameters, and outputs is given in the references [13-16,21].

### Porcine experiments and data

All procedures and protocols used in the porcine experiments were reviewed and approved by the Ethics Committee of the Medical Faculty at the University of Liege (Belgium). Six pure pietrain pigs were premedicated and anesthetized as described previously [23]. Throughout the trials, 10 ml/kg volume-cycle ventilation was provided after endotracheal intubation at a respiratory rate of 20 breaths per minute. Inspired oxygen fraction was set to 40%, and the respiratory settings were adjusted to maintain CO_2 _between 30 and 35 mmHg. Access to the cardiac chambers and pulmonary truck was achieved via median sternotomy. A micromanometer-tipped catheter was inserted into the pulmonary artery through an incision in the right ventricle outflow track and was adjusted to be 2 cm downstream from the pulmonary valve. Another micromanometer-tipped catheter was descended into the thoracic aorta through the femoral artery. Furthermore, 7F, 12-electrode conductance, micromanometer-tipped catheters were positioned in the left and right ventricle so that all electrodes were within these cavities.

To simulate APE, three autologous blood clots of decreasing size (0.25, 0.125, and 0.0625 g kg^-1^) were inserted into the external jugular vein at 0, 120, and 240 minutes into the trials. From the catheters, continuous waveforms over 6 to 12 heartbeats were obtained every 30 minutes (T0 to T270) of left and right ventricular pressures and volumes (*P_lv_*, *P_rv_*, *V_lv_*, *V_rv_*), aortic pressure (*P_ao_*), and pulmonary artery pressure (*P_pa_*). This research uses 46 sets of data from five of the pigs (pig 1, pig 2, pig 3, pig 4, and pig 5) from the study [23]. Measurements from the sixth pig were omitted, because it died very early in the trial.

### Model identification

A new, more clinically applicable, model identification method has been developed to replace the old approach [17-20]. The new method only requires a minimal set of discrete measurements, such as stroke volume and mean aortic pressure, that are easily obtainable in the ICU. Whereas the previous method required measurements of continuous waveforms, including the highly invasive (or expensive) measures of the time varying ventricular volume traces, which require a lot of computation processing. The measurements used by the new approach are far fewer and far less intensive to obtain and utilize. Thus, this study shows a significant step forward in making this type of model-based approach more clinically applicable and accessible without added clinical effort or burden to the patient.

In this research, the model identification method utilizes measurements from the porcine trials to match CVS model to the pig's specific hemodynamics. The process uses *only *clinically available measurements from trials to create the subject-specific models. In this approach, the CVS model acts as a structure of cardiac and circulatory physiology to which model parameters are personalized. Model outputs are matched to a minimal set of measurements, including features from the aortic and pulmonary artery pressure waveforms, stroke volume, heart rate, global end diastolic volume (GEDV), and mitral and tricuspid valve closure times, all of which are commonly available. For this research, GEDV was assumed to be related to the sum of the left and right ventricular end diastolic volumes (LVEDV, RVEDV) calculated from *V_lv _*and *V_rv_*. Clinically, the features required from the aortic pressure waveform, including systolic pressure, diastolic pressure, and maximum pressure gradient, can be inferred from radial artery pressure waveform using methods described previously [26-31]. Furthermore, because only basic features are required from the aortic and pulmonary artery pressure waveforms, high fidelity measurements are not required. Hence, measurements from commonly used fluid-filled catheters with frequency responses greater than 10 Hz can be used with this approach.

The model identification process iteratively compares the outputs from the CVS to corresponding measurements from the minimal clinical data set to find better approximations for the pig-specific parameters. Once the model outputs match the measurement set, the process outputs a CVS model with individualized parameters that describe the pig's specific hemodynamics. A full description of the model identification method can be found in Revie et al. [22] for the interested reader.

### Analysis

For this proof of concept study, the model identification process was tested with 46 sets of porcine data from five pigs with induced pulmonary embolism [23]. Relative percentage errors and accuracy and precision indices derived from absolute errors were calculated. The accuracy indices were derived by calculating the mean of the absolute error of the model outputs for each pig. The precision indices describe the 90^th ^percentile range of the absolute error of the model outputs for each pig. The relative and absolute errors were calculated by comparing the measured LVEDV and REVDV, and maximum left and right ventricular pressures to the model outputs (*P_lv_*, *V_lv_*, *P_rv_*, *V_rv_*). These specific measurements were taken experimentally, but critically, not used to identify the CVS model. Hence, they represent true, independent validation tests of the identified subject-specific model's ability to capture the subject's true dynamics. These comparisons provide a first validation of the subject-specific model's ability to accurately represent the subject at a specific point in time. Second, verification of model convergence is checked by comparing the model outputs to the measured data used in the identification process.

Finally, for proof of concept and clinical relevance, it must be shown that the identified CVS models are capable of tracking clinical metrics of APE as they evolve through time. At each measurement time, the model parameters that are directly related to APE are averaged across the five pigs and compared with the same averaged experimentally derived metric and measurements from Ghuysen et al. [23] to calculate the Pearson correlation coefficient (R^2 ^value). This analysis shows that the identified models (over time) capture clinically relevant and observed diagnostic trends. It would thus, in combination with the other analyses, validate the clinical relevance and concept of this model-based approach.

Please note that due to the small number of subjects (n = 5 with usable measurements) in the study a more in-depth statistical analysis could not be undertaken. Hence, statistically significant changes in the model parameters resulting from the insertion of emboli in the pigs could not be quantified, because the sample size was too small to calculate a clinically meaningful *p *value.

## Results

### Physiological model validation

**Figure 3:**
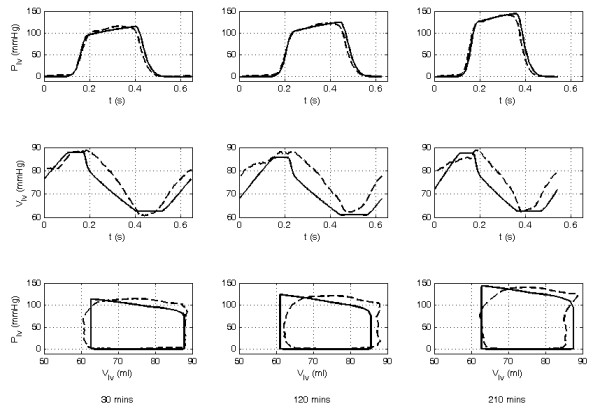
**Comparison of the modeled to measured left ventricular pressure waveform, volume waveform, and pressure-volume loops for pig 4 at 30, 120, and 210 minutes into the trial**. The dashed line shows the measured waveform and the solid line the CVS model output.

**Figure 4:**
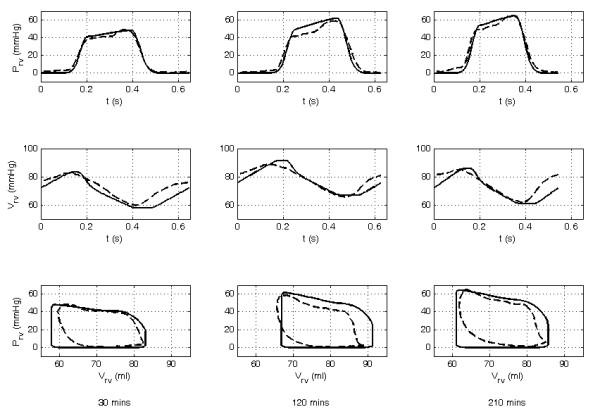
**Comparison of the modeled to measured right ventricular pressure waveform, volume waveform, and pressure-volume loops for pig 4 at 30, 120, and 210 minutes into the trial**. The dashed line shows the measured waveform and the solid line the CVS model output.

Measurements not used in the identification process, were utilized post-identification to validate the accuracy of the pig-specific models. Figures 3 and 4 show an example of the personalised CVS model for pig 4 predicting the measured left and right ventricular volumes and pressures (*P_lv_*, *V_lv_*, *P_rv_*, *V_rv_*) at different time points during the trial. Median and 90^th ^percentile errors were calculated for the modeled mean aortic and pulmonary artery pressures, LVEDV and RVEDV, and the maximum left and right ventricular pressures in each trial as summarized in Table 1. Across all of the pigs, the modeled LVEDV and RVEDV and the maximum left and right ventricle pressures (*P_lv_*, *P_rv_*) lie within absolute error ranges of 4.1% to 15.1%. Furthermore, accuracy and precision metrics were calculated from the absolute errors in the model estimated LVEDV, RVEDV, and maximum *P_lv _*and *P_rv _*values. Noticeably, the errors are largest for pigs 1 and 2. These two pigs died during the trials with large drops in blood pressure and CO observed before death. For example, the mean aortic pressure and CO for pig 1 fall from baseline values of 92.4 mmHg and 2.3 L/min to 37.2 mmHg and 0.7 L/min, respectively. These values fall below what is considered a survivable physiological range for pigs of this size and result in low flow dynamics that cannot be represented by the CVS model. Hence, the averaged errors of Table 1, and the accuracy and precision metrics of Table 2 are distorted by the model outputs identified from the measurement sets recorded directly before death in pigs 1 and 2.

**Table 1:** Median and 90^th ^percentile absolute percentage errors of the model outputs compared to measured data.

Output	Error	Pig 1	Pig 2	Pig 3	Pig 4	Pig 5	All
***P_ao, mean_***	**Median**	0.2	0.1	0.2	0.1	0.1	0.1
	**90^th^**	0.5	0.1	0.4	0.5	0.2	0.4
***P_pa, mean_***	**Median**	0.1	0.1	0.1	0.0	0.1	0.1
	**90^th ^**	0.5	0.1	0.1	0.1	0.1	0.4
**LVEDV**	**Median**	3.3	18.2	4.3	2.5	1.9	4.1
	**90^th^**	15.9	22.8	7.0	6.5	4.6	17.4
**RVEDV**	**Median**	6.5	15.8	4.3	2.6	1.9	4.4
	**90^th^**	14.6	18.9	7.4	7.0	6.9	15.3
***P_lv, max_***	**Median**	11.4	9.2	2.7	1.5	1.7	2.1
	**90^th^**	27.8	23.5	4.5	3.1	2.5	20.5
***P_rv, max_***	**Median**	15.5	30.6	3.7	2.7	18.4	15.1
	**90^th^**	18.3	31.6	15.6	5.7	23.3	27.2

Mean aortic and pulmonary artery pressures (*P_ao, mean _P_pa, mean_*) are used in the identification process, whereas the left and right ventricular volumes (LVEDV, RVEDV), and maximum left and right ventricular pressures, (*P_lv, max_*, *P_rv, max_*) were not for identification and are true validations

**Table 2:** Accuracy and precision of the estimated left and right ventricular volumes (LVEDV, RVEDV), and maximum left and right ventricular pressures (*P_lv, max _P_rv, max_*)

Output	Error	Pig 1	Pig 2	Pig 3	Pig 4	Pig 5
**LVEDV (ml)**	**Accuracy**	-2.1	-11.1	1.2	0.5	-0.8
	**Precision**	8.7	9.6	7.1	5.8	3.5
**RVEDV (ml)**	**Accuracy**	3.9	11.2	0.7	-0.4	2.6
	**Precision**	9.8	9.1	6	5.9	3.5
***P_lv, max _*(mmHg)**	**Accuracy**	-10	-16	3.6	0.8	2.2
	**Precision**	20.9	18.1	4.5	5	3
***P_rv, max _*(mmHg)**	**Accuracy**	8.1	18.7	0.3	1.6	13.6
	**Precision**	4.8	2.4	6.8	3.4	7

### Clinical proof of concept validation

**Figure 5:**
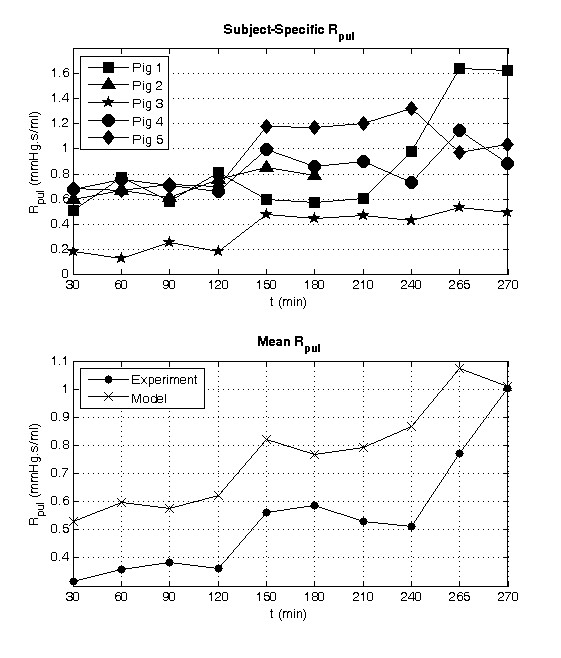
**Identified subject-specific pulmonary resistance (*R_pul_*) during the porcine trials (top) and mean *R_pul _*compared with the experimentally derived pulmonary vascular resistance **[23]**(bottom)**.

The main effect of pulmonary embolism on the CVS is a rise in pulmonary vascular resistance (PVR) or *R_pul _*in the model. Hence, monitoring the opposition to flow in the pulmonary circulation, available only via the model, can give insight into diagnosing and tracking the progress of APE. Although *R_pul _*is not the only component of pulmonary afterload, it is the parameter in the model that is most affected by direct mechanical obstruction to the vasculature and has therefore been chosen for analysis here. In all five trials an increase in *R_pul _*is observed (Figure 5). Averaged over all five pigs, *R_pul _*compared well to a pulmonary vascular resistance derived from a windkessel model [23], which is a standard clinical metric, with a correlation coefficient of R^2 ^= 0.81. Figure 5 shows *R_pul _*systemically overestimating the experimentally derived PVR, but importantly, the modeled value tracks the large increases in PVR at t120 and t240 after pulmonary embolization. However, noticeable discrepancies in the modeled and experimentally derived trends are also visible in Figure 5, especially between T150 and T210 and after T265 where the gradient between the two traces are of opposite slope. These differences result from difficulties in modeling the low flow states, directly before death, in pig 1 (which dies after T270) and pig 2 (which dies after T180). Hence, the averaged *R_pul _*values at T180 and T270 have a larger error associated with them, which account for some of the differences in the slope. Another compounding factor is that after T180 only values from four pigs are averaged instead of five, as pig 2 dies, which causes a slight distortion in modeled *R_pul _*trend at this point.

**Figure 6:**
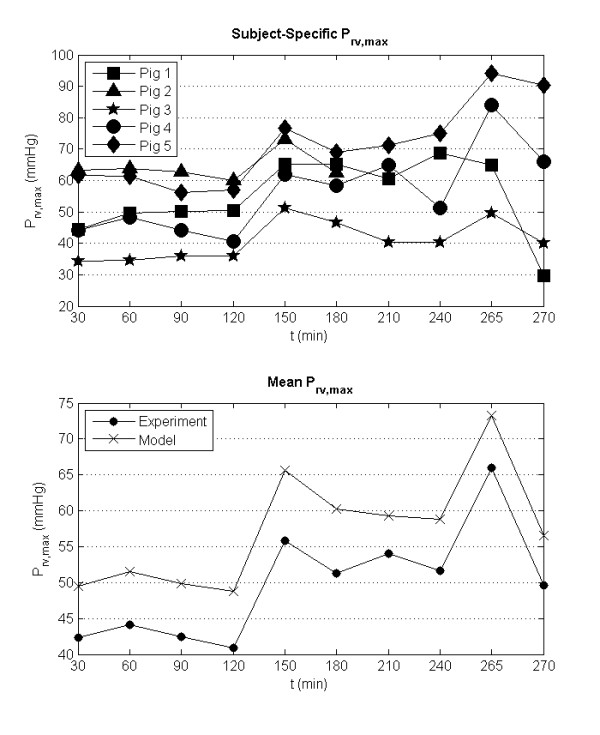
**Identified subject-specific maximum right ventricular pressure (*P_rv, max_*) during the porcine trials (top) and mean *P_rv, max _*compared to the measured maximum right ventricular pressure (bottom)**.

A direct cause of the sharp increase in PVR is acute pulmonary hypertension. Consequentially, the systolic right ventricular pressure must increase to push blood through the pulmonary valve over the ejection cycle. This result is seen in the pig-specific models (Figure 6), where large increases in the maximum right ventricular pressures largely follow changes in *R_pul _*from Figure 5. As with the modeled *R_pul_*, the modeled *P_lv, max _*systematically overestimates the experimental measurements. However, more importantly, the general trend is captured by the model to a high degree of accuracy (R^2 ^= 0.9).

**Figure 7:**
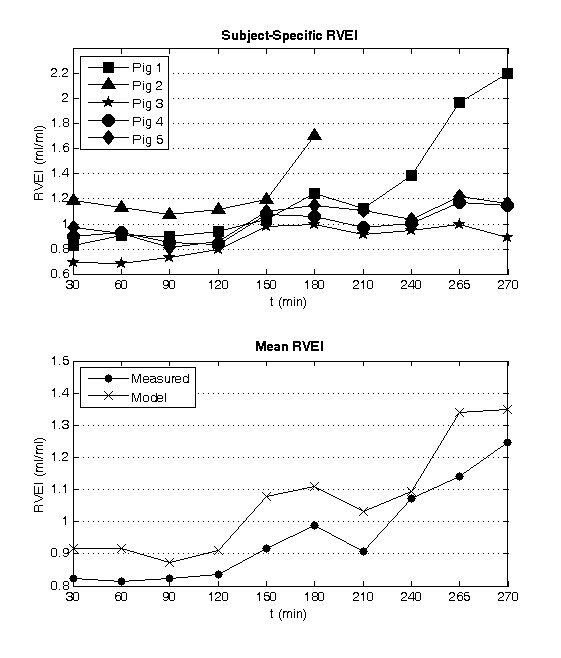
**Identified right ventricular expansion index (RVEI, defined as RVEDV/LVEDV) during the porcine trials (top) and comparison of the modeled to measured mean RVEI (bottom)**.

The right ventricle adapts to increases in pressure by dilating, because it is a compliant chamber. This dilation causes the right ventricle to bulge into the left ventricle resulting in a leftward shift in the intraventricular septum, a known response of APE [32,33]. In the model the right ventricle expansion index (RVEI), which is the ratio of the RVEDV to LVEDV, is used to define the magnitude of this shift. Figure 7 shows the comparison of the modeled and measured averaged RVEI, where the model again overestimates the measured metric but captures the overall trend. Here, the modeled ratio related to the measured RVEI with a correlation coefficient of R^2 ^= 0.92. Interestingly, large increases in RVEI are identified for pigs 1 and 2 that died during the trials.

**Figure 8:**
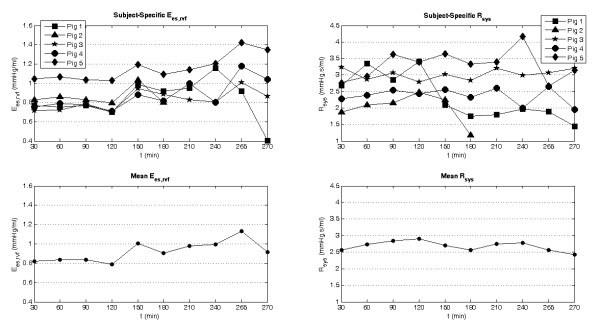
**Identified subject specific right ventricle end systolic elastance (*E_es, rvf_*, top left) and systemic resistance (*R_sys_*, top right) during the porcine trials, along with the mean modeled *E_es, rvf _*(bottom left) and *R_sys _*(bottom right)**.

Of further interest is how each pig's CVS responds to the insertion of emboli. In particular, how the right ventricular contractility, *E_es, rvf_*, reacts to the increased afterload of the pulmonary circulation and how the systemic resistances, *R_sys_*, is altered to maintain the distribution of blood in the CVS. These two reflex responses are shown in Figure 8. The averaged *E_es, rvf _*from the model is seen to increase significantly after the insertion at T120 and T240 and appears to track changes in *R_pul _*from Figure 5. On the other side of the circulation, large drops in *R_sys _*are observed at the same time large increases in RVEI are seen (Figure 7) for pigs 1 and 2, indicating that the loss of systemic vascular tone maybe a contributing factor to the volume imbalance in the ventricles.

## Discussion

### Modeling errors

In response to a pathological challenge, clinically and physiologically model-based hemodynamic metrics typically change by 50-100% and in some cases up to 400%. Nearly all the errors associated with the model outputs are less than 25% (Table 1), indicating that the subject-specific models are identified with sufficient accuracy to identify induced disease states. The larger errors in Table 1 result from differences between the measured left and right ventricle stroke volumes, which are expected to be same at steady state. In some cases, especially pigs 1 and 2, the ratio of the left to right ventricular outputs is greater than 2:1. This discrepancy is probably due to difficulties in measuring the right ventricle volume due to its complex shape, which causes the model to underestimate the end diastolic volumes and maximum pressure of the left ventricle and overestimate the end diastolic volume and maximum pressure of the right ventricle. However, the ratio of the measured stroke volumes is generally consistent for each pig for the duration of the trial resulting in systematic errors in the model outputs, so importantly, changes in the model trends are preserved.

The variation in shape and difference in phase of the modeled outputs compared with the measured waveforms (Figures 3 and 4) are due to the lumped parameter nature of the model and errors in the approximated driver functions (*driL *and *driR*) used to describe the myocardial contraction.

### Detecting pulmonary embolism

The main hemodynamic consequence of APE is a blockage in the pulmonary vasculature leading to the lungs, resulting in increased pulmonary afterload. Afterload is a combination of the simple relationship between the mean arterial pressure and total flow across the system and the more complex relationships due to the pulsatile and wave reflective nature of circulatory flow. In addition, in the pulmonary circulation, afterload has an added component due gravitational effects in lung and the collapsible nature of alveoli capillaries. It has been recognized in recent times that pulmonary vascular resistance (PVR which is the same as *R_pul_*) is not the ideal measure of pulmonary afterload [34,35], because it does not consider all of these components of afterload. Furthermore, *R_pul _*in the model is calculated by assuming pulmonary vein pressure is the downstream pressure, although may not be the case in certain scenarios. However, PVR is still the most widely used clinical metric of pulmonary afterload due to its relative ease of measurement and lack of widely accepted alternative. Hence, in this research *R_pul _*was used to track changes in pulmonary afterload resulting from the inserted emboli.

Through identifying subject-specific models for each pig, pulmonary vascular resistance (*R_pul_*) was tracked. Figure 5 shows the predicted *R_pul _*for each pig across the duration of the trials. In all five pigs, *R_pul _*increased during the trials, indicating the presence of a pulmonary vascular obstruction, as expected. However, note that *R_pul _*drops for most pigs at the end of the trials as CO begins to fall significantly.

Figure 5 shows the model tracking changes in the experimentally derived mean pulmonary vascular resistance calculated for the pigs using a four element windkessel model [23]. The model appears to systemically produce larger resistances compared to the experimentally derived parameters. However, more importantly, the models track the averaged experimental trends with a correlation of R^2 ^= 0.81. The model also reveals sharp increases in pulmonary vascular resistance caused from the insertion of autologous blood clots at 120 and 240 minutes into the trials, as expected and illustrated in Figure 5. These results indicate this approach can capture clinically relevant change in pulmonary vascular resistance, a major indicator of APE.

### Autonomous reflex responses

The condition of a critically ill patient can be determined by how well their autonomous cardiovascular reflex responses control circulatory state. In the porcine trials, each pig's condition can be analyzed through how effectively their CVS adapts to the pulmonary embolism or emboli. One consequence of a blockage in the pulmonary vasculature is an accumulation of blood upstream of the obstacle resulting in less flow to left heart. To compensate, the CVS responds in two main ways: 1) by increasing systemic vascular resistance, *R_sys _*[36]; and 2) by strengthening the contractility of the right heart, *E_es_,_rvf _*[25].

In the model, *R_sys _*increases to stop flow leaving the systemic circulation to maintain preload on the left ventricle, whereas *E_es, rvf _*rises to push blood through the more resistant pulmonary system to avoid accumulation of volume upstream of the embolism and to prevent the right heart from overdistending. Figure 8 shows the modeled *R_sys _*increasing initially followed by a sudden decrease for pigs 1 and 2, indicating a loss of autonomous control. The model also captures sharp increases in right ventricle contractility (*E_es, rvf_*) after the insertion of autologous blood clots at 120 and 240 minutes into the trials (Figure 8). This latter response indicates that the pigs autonomously maintain hemodynamic stability. However, failure of *E_es, rvf _*to fully counteract increases in *R_pul _*has an eventual negative impact, as seen at the end of the pig 1 trial, where a large drop CO is noticed as *E_es, rvf _*drops dramatically. These two examples show how this model-based approach can be used to reveal the magnitude of the subject's natural response to the emboli that can help distinguish how the subject is coping with the disease state. In a clinical environment, these parameters also could be monitored to observe what effect drugs, such as vasopressors and inotropes, are having on the patient.

### Severity of pulmonary embolism

The joint response of *E_es, rvf _*and *R_sys _*attempts to prevent a leftward shift in the intraventricular septum wall, which would reduce the stressed volume in the left ventricle and therefore compromise left heart function. The right ventricle expansion index (RVEI), defined as the ratio of the right to left end diastolic volumes, acts as an indicator of how well an individual's autonomous reflex responses are handling the effects of APE [19,32,33]. When the reflex responses can no longer preserve homeostasis in the CVS, the RVEI will increase dramatically as the intraventricular septum pushes into the left ventricle volume. The preload on the left ventricle will be reduced and CO will drop as a result [37]. Hence, RVEI describes how well the subject's natural reflexes are counteracting the detrimental effects of APE.

Averaged across all five pigs, the identified RVEI compared well with the true RVEI with a correlation coefficient of R^2 ^= 0.92. Figure 7 shows that the modeled RVEI increases in all the trials, with sharp increases noticed for pigs 1 and 2 near the end of their trials, indicating that they are in a near-death state. Whereas only minor increases (< 25%) are noticed for pigs 3, 4, and 5, which survive the trials. Hence, the modeled RVEI provides a good indication of how well the pigs are coping with APE.

### Limitations

The main limitation of model identification method is its reliance on knowing the pulmonary artery pressure, which is not widely measured. The use of the pulmonary artery catheter (PAC) has gone out of fashion in recent times, because it has been found to have little or even deleterious effects on mortality rates [38-41]. Some investigators [41] have suggested the use of the PAC by itself may not be the problem. Instead, incorrect interpretation of the waveform and the use of suboptimal therapies triggered from data obtained from the PAC may be the cause of the poorer outcomes. In such cases, patient-specific models could be used to standardize interpretation of the measurement. Features of the pulmonary artery waveform in conjunction with other measurements can be converted into easy to understand metrics representing ventricular contractility, preload, vascular stiffness, and resistance, which are directly controllable with current clinical treatments (fluid resuscitation, inotropes, vasopressors, and vasodilators), providing a better platform for therapy to be based from. Given the potential, and only small added risks associated with the PAC [42-47], this compromise favors improved patient outcome, as well as ease of use. Alternatively, the model identification method could easily be adapted to use echocardiography measurements to identify parameters of the pulmonary circulation, although this would reduce the frequency at which the CVS models could be identified.

Other limitations of this technique are due to the lumped parameter nature of the computer CVS model and the limited number measurements typically available in the ICU. The lumped parameter nature of the model restricts this technique from identifying highly localized changes along the circulation. However, as seen with APE, the effect of a major localized dysfunction normally manifest itself on the whole circulation and therefore can be identified.

The small number of measurements available in the ICU restricts the number of parameters that can be identified. Therefore, slow changing parameters, or parameters that have lesser effect on the circulation in the computer model, are held constant so that changes in more physiologically relevant and sensitive parameters can be tracked. For example, in the model the left ventricle zero pressure volume (*V_0, lvf_*) is not identified and is fixed as it remains relatively constant in the absence of major inotropic or loading interventions. By fixing *V_0, lvf _*the more important end systolic left ventricle elastance (*E_es, lvf_*) can be identified and changes in this parameter can be tracked. Finally, although this process is restrictive, it enables an approach that requires no added invasive measurements.

## Conclusions

The computer models of the CVS correctly estimated the known physiological responses to APE. An increase in *R_pul_*, the main hemodynamic consequence of APE, related well to the experimentally derived pulmonary vascular resistance. The individualized models also captured a loss of autonomous control of important reflex responses, *R_sys _*and *E_es, rvf_*, in pigs 1 and 2, leading to cardiovascular failure. Furthermore, the identified RVEI increased as emboli were inserted into the pigs, indicating a leftward shift in the intraventricular septum, which accurately matched the changes in the measured RVEI. These results clearly show that subject-specific models can be used to identify clinically useful and relevant cardiac and circulatory information in acute CVS dysfunction by accurate regular assessment of (model-based) subject-specific CVS status. Hence, this research demonstrates and proves the clinical relevance, concept, and potential of this model-based approach to CVS monitoring and diagnosis.

## Abbreviations

APE: acute pulmonary embolism; CO: cardiac output; CVS: cardiovascular system; *E_es, rvf_*: modeled right ventricular end systolic elastance; GEDV: global end diastolic volume; ICU: intensive care unit; LVEDV: left ventricular end diastolic volume; *P_ao_*: aortic pressure waveform; *P_lv_*: left ventricular pressure waveform; *P_lv, max_*: maximum left ventricular pressure; *P_pa_*: pulmonary artery pressure waveform; *P_rv_*: right ventricular pressure waveform; PVR: pulmonary vascular resistance; *R_pul_*: modeled pulmonary vascular resistance; *R_sys_*: modeled systemic vascular resistance; RVEDV: right ventricular end diastolic volume; RVEI: right ventricular expansion index; *V_lv_*: left ventricular volume waveform; *V_rv_*: right ventricular volume waveform.

## Competing interests

The authors declare that they have no competing interests.

## Authors' contributions

JAR developed and adapted the computer-based CVS monitoring system for use with animal hemodynamic data and contributed to the analysis of the results. DJS created the method for subject-specific identification of time varying elastance and contributed to the development of the identification of the CVS model parameters. JGC substantially contributed to the analysis and interpretation of the results. CEH developed the general method to identify CVS model parameters. BCL contributed to the development and implementation of the animal model of acute pulmonary embolism and assisted with the analysis of results. AG, PK, and PM contributed to the development and implementation of the animal model of acute pulmonary embolism. GMS assisted with the clinically relevant parts of the analysis. TD contributed to the analysis of the results in the paper and assisted with developing the animal model of acute pulmonary embolism.
